# Takotsubo Cardiomyopathy: Patients Characteristics, Mortality, and Clinical Significance of Left Ventricular Outflow Tract Gradient, Retrospective Study

**DOI:** 10.1155/2024/5549795

**Published:** 2024-07-09

**Authors:** Yunis Daralammouri, Hamza Hamayel, Dina Abugaber, Sari Nabulsi

**Affiliations:** ^1^ Department of Cardiology An-Najah National University Hospital, Nablus, State of Palestine; ^2^ Department of Medicine Faculty of Medicine and Health Sciences An-Najah National University, Nablus, State of Palestine; ^3^ Department of Critical Care An-Najah National University Hospital, Nablus, State of Palestine; ^4^ Department of Medicine An-Najah National University Hospital, Nablus, State of Palestine

## Abstract

**Background:**

Takotsubo cardiomyopathy (TC) is a reversible left ventricular systolic dysfunction with apical ballooning. Left ventricular outflow tract (LVOT) obstruction may develop in these cases due to hyperdynamic state of the left ventricle. Limited data are available on the prevalence of LVOT gradient in TC and its association with patient outcomes and mortality.

**Methods:**

Data were collected retrospectively for patients diagnosed with TC in a single tertiary center, demographic information, blood analysis results, and imaging finding including ejection fraction, and LVOT gradient was obtained from medical records. Additionally, medical treatment and outcome during hospitalization were extracted. Follow-up was conducted through cardiology clinic or phone contact.

**Result:**

A total of 59 patients diagnosed with TC were reviewed during hospitalization, and 40 patients were followed up after discharge by phone contact and cardiology clinic. Most patients were female (91.5%), and nonsignificant coronary artery disease was present in 16.9% of patients. Approximately two-third of the patients had a reduced ejection fraction on admission, and only two patients (5.4%) continued to have reduced ejection fraction on echocardiography follow-up within a period of 2–14 days. LVOT gradient was present in 17 patients (28.5%); patients with preserved ejection fraction had a higher probability of having an LVOT gradient. However, there was no association between LVOT gradient and shock or mortality. Four patients (6.7%) experienced 30-day mortality, while all-cause mortality was reported in eight patients (13.5%) over the follow-up period (mean (±SD) 20.8 months ± 16.2).

**Conclusion:**

LVOT obstruction may occur in TC patients; it has no correlation with shock or mortality. However, determining whether there is a gradient is important for deciding on specific treatment approach.

## 1. Background

Takotsubo cardiomyopathy (TC), also known as apical ballooning syndrome, is a specific form of nonischemic cardiomyopathy [1]. It typically occurs after emotional or physical stress although no trigger could be identified in many patients [2]. The disease's pathophysiology is still unknown. However, many features of the disease suggest its relation to microvascular spasm or dysfunction caused by catecholamine excess associated with emotional or physical stress [3].

The prevalence of the disease is increasing, and it mostly affects women, with postmenopausal women making up the majority [4]. TC represents around 2–3% of patients who present as acute coronary syndrome (ACS) [2]. Although TC typically has no significant coronary artery disease (CAD), initial workup includes troponin, electrocardiogram (ECG), transthoracic echocardiography (TTE), and cardiac catheterization to rule out significant CAD as most of these patients present initially with chest pain and shortness of breath [5–7].

Transient regional systolic dysfunction is characteristic of this type [8], mainly involving the ventricular apex, and less likely mid ventricle or base of the heart (inverted type) [2, 9]. This is evident from the finding on left ventriculography through cardiac catheterization and echocardiography image [2].

Since it is usually transient and resolves with time, the prognosis is generally favorable. However, numerous complications may arise during acute presentation [10, 11]. Approximately 20% of patients experienced serious adverse events during hospitalization, which is comparable to patients with acute coronary syndrome [12]. The mortality rate during hospitalization is reported to be around 1–5% [12–14], while long-term mortality found to be about 5–6% per patient-year [15, 16].

Left ventricular outflow tract (LVOT) gradient has been reported in about 10–25% of patients with TC [17], hypothesized from the result of apical myocardial stunning and hypercontractility of the basal segment as a compensatory mechanism [14]. The presence of LVOT gradient in patients with TC and its association with other variables and outcomes is not well studied [14, 18]. In our article, we describe the characteristics and findings of patients with TC in a single tertiary center, focusing on its association with LVOT gradient and its impact on mortality.

## 2. Methods

### 2.1. Study Design and Population

This is a retrospective cohort study of patients diagnosed with TC. Data were collected from a single tertiary center, representing patients admitted to the hospital as a case of ACS, and later on diagnosed as TC over a period of six years. Upon admission, demographic data were collected, diagnostic workup including serum troponin, ECG, and TTE was performed, and all patients underwent cardiac catheterization to rule out significant CAD.

All patients diagnosed with TC were enrolled in this study. The diagnosis of TC was based on Mayo criteria, which state that TC is diagnosed by the presence of new ECG changes or modest elevation of troponin, transient left ventricular dysfunction, no significant CAD, and no pheochromocytoma or myocarditis [19].

Baseline demographic data, clinical profile, ECG, echocardiography, and coronary angiograph parameters including LVOT gradient were extracted from medical records. Medical treatment and outcomes during hospitalization were also retrospectively collected. Follow up after discharge was conducted via phone contact.

Cardiac catheterization was done within 48 hours of presentation. After excluding significant CAD, left ventriculography was performed revealing apical ballooning of the left ventricle. The gradient between apex and the aorta was then calculated using pullback method of the pigtail to measure peak-peak LVOT gradient, considered positive if peak-peak LVOT gradient ≥20 mmHg [14].

TTE was performed on the day of admission, and repeated after a few days of hospitalization with documentation of ejection fraction using Simpson method. According to the clinical picture and finding on TTE and cardiac catheterization, further diagnostic workups such as cardiovascular magnetic resonance (CMR) and endomyocardial biopsy were considered.

### 2.2. Ethical Considerations

The institutional review board (IRB) granted ethical approval. Each document of information was kept private and maintained in a closed storage cabinet within a secure office. The original data forms were accessible to the principal investigator and coinvestigator. All serious or unexpected adverse events that might affect the safety of research participants were to be reported by the principal investigator.

### 2.3. Statistical Analysis

The statistical package of social science (SPSS) was used for data analysis in this study. Demographic characteristics of the sample are presented using tables and/or figures as appropriate. The percentage of each study variable and the success rate were calculated. The significant difference between groups was assessed using the chi-squared test, Fisher exact test, and Mann–Whitney *U* test, as appropriate, and log rank test was used for survival analysis. The difference was considered significant when the *P* value is ≤0.05.

## 3. Result

### 3.1. Baseline Characteristic of Patients

Our data describe characteristics of 59 patients admitted were admitted to the center and diagnosed with the apical type of TC. Baseline characteristics of the patients are described in Table 1. Most patients were elderly (mean (±SD), 72.4 ± 9.8 years), with females constituting the majority of patients (91.5%), particularly postmenopausal women. About 16.9% of patients had coronary artery disease (which was not significant), and other comorbidities such as hypertension and type II diabetes mellitus were reported as following 69.5% and 27.1%, respectively.

### 3.2. Clinical Presentation, Medical Treatment, and Outcomes for Patients with TC

Table 2 describes the clinical presentation of patients with TC. More than half of the patients presented with chest pain (57.6%), followed by dyspnea (40.7%) and rarely they present with syncope (3.4%). A Trigger could not be identified in around one-third of patients (37.3%). The remaining patients had triggers either physical or emotional stress. The reported physical stress were infections, severe pain, and postoperative condition including pacemaker insertion. Workup on admission showed abnormal ECG changes evidenced by ST-T changes or T wave inversion in 76.3% of patients, around 7% of patients had atrial fibrillation, and troponin was positive in almost all patients (96%). Peak-peak LVOT gradient was measured in all patients, and was present in 17 (28.5%) patients, it ranges from 20 mmHg to 120 mmHg (mean (±SD), 32.5 mmHg ± 26.6). Another patient had no gradient at presentation and increased after recovery of left ventricular function [20]. On the day of admission, TTE's evaluation of ejection fraction was (mean (±SD), 38.8% ± 8.9), revealing a reduced ejection fraction (≤40%) in 35 patients (64.8%), while improvement in ventricular function was seen in almost all patients within few days (mean (±SD), 5.2 days ± 3.34). For patients with documented reports for reevaluation of left ventricular function within this period (37 patients), all had improved ejection fraction (mean (±SD) 55.7 ± 7.4), and only two of them (5.4%) remained in the reduced ejection fraction group.

During hospitalization, shock was present in 20.3% of patients; all patients were given supportive treatment, and other medications including angiotensin converting enzyme inhibitor (ACE-I) and beta blocker (BB) were prescribed in most of the patients 84.4%, 89.6%, respectively, either on admission or after shock resolution. The 30 days mortality rate was 4 patients (6.7%), and all-cause mortality during follow-up period which ranges from 1 to 48 months (mean (±SD) 20.8 months ± 16.2) was 8 patients (13.5%). Malignant arrhythmia (ventricular tachycardiac and ventricular fibrillation) was reported in 4 patients (6.7%).

### 3.3. Association between LVOT Gradient with Different Variables

There was no association between LVOT gradient and most of variables as shown in Table 3. Patients with preserved ejection fraction on presentation had statistically significant association with presence of LVOT gradient compared with patients who had reduced ejection fraction (47.4% vs. 22.9%, *P*=0.05).

**Figure 1:**
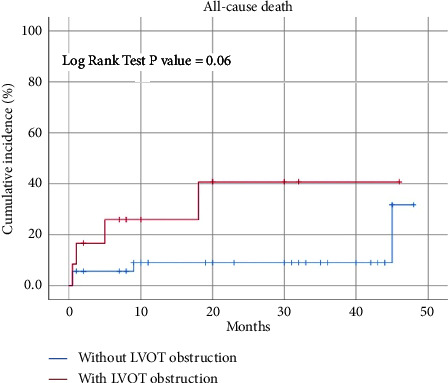
Kaplan–Meier curve of Takotsubo cardiomyopathy (TC) represent all-cause death in patients with and without left ventricular outflow tract obstruction.

When mortality assessed, there was no statistically significant association between neither presence of LVOT gradient nor severity of LVOT gradient and mortality as shown in Figure 1. Mortality was also not associated with troponin, underlying trigger, medical treatment during hospitalization, and reduced ejection fraction. However, patients with chest pain have lower rate of mortality than patients who did not have pain on presentation (3.3% vs. 38.9%, *P*=0.001), and of course, presence of shock had significant statistical association with mortality as shown in Table 4.

## 4. Discussion

Our study represents the characteristic of patients diagnosed with TC. Age and sex were not distributed equally, the majority of patients were female (91%) and elderly (mean age 72 years), which is consistent with reported data in literature [4, 20]. Large registry data worldwide shows varying percentage of female's percentage in different population, ranging from 78% in Japan's population to 91% in USA population [21]. Additionally, older age was more prevalent similar to different large registry data with the mean age in sixties and seventies [22]. The underlying mechanism for this difference is unclear, declining in estrogen level is one of the possible theories, but it cannot explain the occurrence of disease in male [21].

Clinical presentation of TC is very similar to ACS [12], and around 81.4% of patients have chest pain or dyspnea, troponin is positive in 96.3%, and ECG changes occur in 76.3%. This supports the importance of early cardiac catheterization to exclude significant CAD. On the other hand, the presence of nonsignificant CAD does not exclude diagnosis of TC, which was seen in 16.9% of our patients, and it has no prognostic factor [23].

According to earlier studies, emotional triggers were the primary reported factors preceding TC; subsequent reports included other trigger factors, such as physical stress, and the absence of a trigger was also reported [2, 24]. In our study, emotional triggers and the absence of triggers were more common preceding TC followed by physical triggers. There is heterogenicity in the literature regarding the main evident factor [24], this difference mostly related to different population and varying identification of specific triggers. These factors were not found to have a prognostic effect on the outcome of patients, in contrast to other studies which showed that emotional triggers have a good prognostic factor [24–26]; this could be due to inappropriate identification of the trigger or due to small sample size.

**Figure 2:**
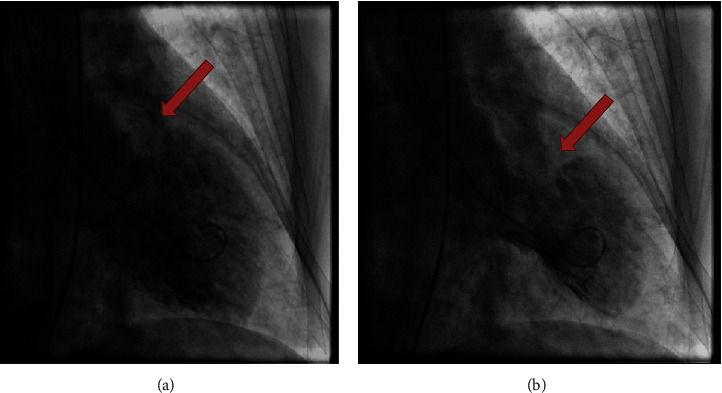
This is right anterior oblique view for a patient with Takotsubo cardiomyopathy. Diastole in the (a) and systole in the (b). It shows hypercontractility of the basal segment evident by round flask and short neck (arrow) at end systole, while there is hypokinesia/akinesia in the apical portion of the heart.

LVOT gradient is present in about 10–25% of patients with TC. The left ventricle experiences a paradoxical reaction from excess catecholamine, resulting in stunning of the apex and hypercontractility at the basal region as shown in Figure 2; this effect together with myocardial edema contributes to increased blood flow through LVOT resulting in gradient development in TC mainly in the apical type form [27]. This is unlike hypertrophic obstructive cardiomyopathy (HOCM) which is caused by septal hypertrophy of the left ventricle [28]. This gradient can be hidden in the presence of reduced ejection fraction due to low flow state [29]. This is consistent with our finding that patients with preserved EF had a higher prevalence of LVOT gradient than patients with reduced EF (47.4% vs 22.9%, *P* value 0.05).

Cardiogenic shock reported in about 20% of patients, and severe systolic dysfunction or significant LVOT gradient could be the main predominant feature. Although there is no statistically significant association between LVOT gradient and cardiogenic shock, a significant LVOT gradient could exist. Understanding the underlying mechanism of shock is crucial in the management plan. Supportive treatment and treating potential trigger factor are recommended for all patients; further treatment depends on the mechanism of underlying shock. In the presence of significant LVOT gradient, left ventricular filling is important to decrease obstructive effect and improve cardiac output. For this, fluid administration is recommended and beta blocker is considered. On the other hand, diuretics as well as vasodilator and inotropic agent should be avoided, as they may worsen LVOT gradient and aggravate cardiogenic shock in patients with significant LVOT gradient [5, 14, 27, 30]. In both cases, mechanical circulatory support may be used as bridging therapy until the disease resolves [14, 27].

30 day mortality rate in TC is reported around 4% [31], and this is very close to our result, which reports 6.7%, similar to STEMI patients [31]. While long-term mortality rate ranges from 5 to 6% per patient-year [15, 16], which is higher than MI patients, this mostly due to the older age of patients with TC compared to MI patients. The presence of LVOT gradient has numerically higher rate of mortality compared to patients without LVOT gradient (30.5% vs 11.4%) as shown in Kaplan–Meier curve. However, this association was not statistically significant. This is also found in similar studies suggesting that gradient has no prognostic factor on mortality [14, 18]. On the other hand, it is important to measure the gradient in all patients to guide in the treatment as we mentioned.

According to published research, the presence of chest pain, emotional trigger, young age, and female are all favorable prognostic factors [10]. Our result found a similar association with chest pain, while there was no association with other factors, possibly due to small sample size. Additionally, BB and ACE-I have no protective effect on mortality, which is also reported in literature [30]. This could be because of the nature of the disease itself, which is temporary and reversible even without medications.

There were many limitations in the study. It is a retrospective study, we depend on the reported information in the medical records, and missing data were a problem. Additionally, it was conducted in a single center, for which, the sample size is small and some association cannot be made, and we cannot generalize this finding due to the different treatment strategies in different hospitals.

## 5. Conclusion

Presence of LVOT gradient among patients with TC is an interesting finding, and our study showed no significant association between LVOT gradient in TC patients and mortality. However, determining the presence of an LVOT gradient will help in guiding specific management plan.

## Figures and Tables

**Table 1:** Baseline characteristics of patients with Takotsubo cardiomyopathy.

Characteristics	Total
Age (mean ± SD)	72.4 ± 9.8

*Gender*	
Male	5 (8.5)
Female	54 (91.5)

*CAD*	
Yes	10 (16.9)
No	49 (83.1)

*Diabetes*	
Yes	16 (27.1)
No	43 (72.9)

*Hypertension*	
Yes	41 (69.5)
No	18 (30.5)

SD: standard deviation, CAD: coronary artery disease.

**Table 2:** Clinical presentation, treatment, and outcome.

Characteristics	Total
*Pain*	
Yes	34 (57.6)
No	25 (42.4)

*Dyspnea*	
Yes	24 (40.7)
No	35 (59.3)

*Trigger*	
Unknown	22 (37.3)
Emotional	22 (37.3)
Physical	15 (25.4)

*Troponin*	
Positive	51 (96.23)
Negative	2 (3.77)

*ECG changes*	
Yes	45 (76.3)
No	14 (23.7)

*Atrial fibrillation*	
Yes	4 (6.9)
No	54 (93.1)

*Shock*	
Yes	12 (20.3)
No	47 (79.7)

*LVOT*	
Present	17 (28.5)
Absent	42 (71.2)

EF (mean ± SD)	38.88% ± 8.9

*Reduced EF*	
Yes	35 (64.8)
No	19 (35.2)

*Reduced EF on follow up*	
Yes	2 (5.4)
No	35 (94.6)

*Beta blocker*	
Yes	52 (89.66)
No	6 (10.34)

*ACE-I*	
Yes	49 (84.48)
No	9 (15.52)

SD: standard deviation, ECG: electrocardiogram, LVOT: left ventricular outflow tract, EF: ejection fraction, ACE-I: angiotensin converting enzyme inhibitor.

**Table 3:** LVOT gradient in relation to baseline characteristics and clinical presentation.

Characteristic	LVOT gradient		*P* value^*∗*^
	No (42)	Yes (17)	
*Gender*			
Male	4 (80%)	1 (20%)	0.64
Female	38 (70.4%)	16 (29.6%)	

*Age*	75.2 ± 6.3	71.3 ± 10.5	0.26^*∗∗*^

*CAD*			
Yes	7 (70%)	3 (30%)	0.92
No	35 (71.4%)	14 (28.6%)	

*Diabetes*			
Yes	11 (68.8%)	5 (31.3%)	0.80
No	31 (72.1%)	12 (27.9%)	

*Hypertension*			
Yes	29 (70.7%)	12 (29.3%)	0.90
No	13 (72.2%)	5 (27.8%)	

*Trigger*			
Unknown	17 (77.3%)	5 (22.7%)	0.51
Emotional	16 (72.2%)	6 (27.3%)	
Physical	9 (60%)	6 (40%)	

*Troponin*			
Positive	35 (68.6%)	16 (31.4%)	0.49
Negative	2 (100%)	0 (0.00%)	

*Shock*			
Yes	6 (50%)	6 (50%)	0.06
No	36 (76.6%)	11 (23.4%)	

*Beta blocker*			
Yes	36 (69.2%)	16 (30.8%)	0.62
No	5 (83.3%)	1 (16.7%)	

*ACE-I*			
Yes	36 (73.5%)	13 (26.5%)	0.44
No	5 (55.6%)	4 (44.4%)	

*Dyspnea*			
Yes	15 (62.5%)	9 (37.5%)	0.22
No	27 (77.1%)	8 (22.9%)	

*Reduced EF*			
Yes	27 (77.1%)	8 (22.9%)	0.05
No	10 (52.6%)	9 (47.4%)	

*Pain*			
Yes	27 (79.4%)	7 (20.6%)	0.10
No	15 (60%)	10 (40%)	

*Atrial fibrillation*			
Yes	3 (75%)	1 (25%)	0.79
No	38 (70.4%)	16 (29.6%)	

*ECG changes*			
Yes	31 (68.9%)	14(31.1%)	0.48
No	11 (78.6%)	3 (21.4%)	

SD: standard deviation, ECG: electrocardiogram, LVOT: left ventricular outflow tract, EF: ejection fraction, ACE-I: angiotensin converting enzyme inhibitor, CAD: coronary artery disease. ^*∗*^Fisher exact test, ^*∗∗*^Mann–Whitney *U* test.

**Table 4:** Mortality in relation to baseline chrematistics and clinical presentation.

Characteristic	Mortality		*P* value^*∗*^
	No (40)	Yes (8)	
*Gender*			
Male	1 (50%)	1 (50%)	0.19
Female	39 (84.8%)	7 (15.2%)	

Age	72.3 ± 10.3	68.3 ± 6.6	0.14^*∗∗*^

*CAD*			
Yes	7 (87.5%)	1 (12.5%)	0.72
No	33 (82.5%)	7 (18.5%)	

*Diabetes*			
Yes	11 (84.6%)	2 (15.4%)	0.88
No	29 (82.9%)	6 (17.1%)	

*Hypertension*			
Yes	30 (88.2%)	4 (11.8%)	0.15
No	10 (71.4%)	4 (28.6%)	

*Trigger*			
Unknown	17 (89.5%)	2 (10.5%)	0.65
Emotional	15 (78.9%)	4 (21.1%)	
Physical	8 (80%)	2 (20%)	

*Troponin*			
Positive	35 (83.3%)	7 (12.7%)	0.30
Negative	1 (50%)	1 (50%)	

*Shock*			
Yes	3 (33.3%)	6 (66.7%)	≤0.001
No	37 (94.9%)	2 (5.1%)	

*Presence of LVOT*			
Yes	9 (69.2%)	4 (30.8%)	0.11
No	31 (88.6%)	4 (11.4%)	

*Beta blocker*			
Yes	37 (86%)	6 (14%)	0.16
No	2 (50%)	2 (50%)	

*ACE-I*			
Yes	35 (89.7%)	5 (10.3%)	0.12
No	4 (57.1%)	3 (42.9%)	

*Dyspnea*			
Yes	14 (73.7%)	5 (26.3%)	0.14
No	26 (89.7%)	3 (10.3%)	

*Pain*			
Yes	29 (96.7%)	1 (3.3%)	≤0.001
No	11 (61.1%)	7 (38.9%)	

*Reduced ejection fraction*			
Yes	23 (76.7%)	7 (23.3%)	0.26
No	14 (93.3%)	1 (6.6%)	

*ECG changes*			
Yes	32 (82.1%)	7 (17.9%)	0.62
No	8 (88.9%)	1 (11.1%)	

SD: standard deviation, ECG: electrocardiogram, LVOT: left ventricular outflow tract, EF: ejection fraction, ACE-I: angiotensin converting enzyme inhibitor, CAD: coronary artery disease. ^*∗*^Fisher exact test, ^*∗∗*^Mann–Whitney *U* test.

## Data Availability

All data supporting the study are presented in the manuscript or available upon request from the corresponding author of this manuscript.

## Abbreviations

TC: Takotsubo cardiomyopathy
ACS: Acute coronary syndrome
TTE: Transthoracic echocardiography
ECG: Electrocardiogram
CAD: Coronary artery disease
LVOT: Left ventricular outflow tract
CMR: Cardiovascular magnetic resonance
ACE-I: Angiotensin converting enzyme inhibitor
BB: Beta blocker
EF: Ejection fraction
DM: Diabetes mellitus
STEMI: ST elevation myocardial infarction
HOCM: Hypertrophic obstructive cardiomyopathy
MI: Myocardial infarction.
